# Bacterial Communities in the Endophyte and Rhizosphere of White Radish (*Raphanus sativus*) in Different Compartments and Growth Conditions

**DOI:** 10.3389/fmicb.2022.900779

**Published:** 2022-06-29

**Authors:** Nan Sun, Yizhu Gu, Guoxia Jiang, Yuxin Wang, Pingzhi Wang, Weitang Song, Peifang Ma, Yabin Duan, Ziyuan Jiao

**Affiliations:** ^1^College of Water Resources & Civil Engineering, China Agricultural University, Beijing, China; ^2^Henan Pingdingshan Academy of Agricultural Sciences, Pingdingshan, China

**Keywords:** ecological compartment, endophyte, phylloplane, rhizosphere, white radish, co-occurrence networks

## Abstract

Endophyte resources have important research value in multiresistance breeding, ecological protection, germicide development, and other fields. In this study, high-throughput sequencing (Illumina-MiSeq) technology was employed to analyse the diversity and community composition of white radish (*Raphanus sativus*) endophytes and rhizosphere bacteria in different compartments and cultivation conditions, including greenhouse and open field cultivation, at both the phylum and genus levels. Alpha diversity index analysis showed that the bacterial richness and diversity values of rhizosphere bacteria were higher than those of endophytes in different compartments. NMDS analysis and microbial co-occurrence network analysis showed that apart from the similarity in the endophytic bacterial composition of the leaf and root endosphere, the endophytic bacterial composition in flesh and epidermis of radish were also more similar. The dominant endophytic bacteria in white radish were Proteobacteria, Bacteroidetes, and Actinomycetes at the phylum level. We analyzed the effects of different ecological compartments and two cultivation environments on radish microorganisms, and found that ecological compartments played an important role, which was related to the mechanism of microbial assembly in plants. The same facility cultivation can also improve the diversity of radish microorganisms in different ecological compartments, and change the biomarkers that play a major role in rhizosphere microorganisms and endophytes of radish. Bacteria plays an important role in the process of plant growth, and the study of endophytes enriches the understanding of microbial diversity in white radish, which helps to provide insight into the ecological function and interaction mechanisms of plants and microorganisms.

## 1. Introduction

In recent years, research on the interaction between plants and microorganisms has increased gradually, but the understanding of the ecological function of plant microorganisms is still limited, especially from the perspective of crop breeding for disease resistance and cold tolerance. Plant endophytes are ubiquitous in the cells of organs and tissue such as roots, stems, leaves, flowers and fruits and have maintained a certain close relationship with host plants during their long evolutionary process. Most plant endophytes are beneficial to plants and can produce biologically active substances that play an intriguing role in the microbe-host relationship. Different endophytes occupy different compartments, and their interactions reach a dynamic balance.

Studies have shown that the distribution of plant endophytes is affected by many factors, such as plant species, geographic location, growth environment, and climatic conditions. Therefore, endophytes also have rich biodiversity (Long et al., 2015). Wang et al. (2006) found *Pantoea*, *Erwinia*, *Salmonella*, *Enterobacter*, *Citrobacter*, *Klebsiella*, and other endogenous bacteria on legumes in Mexico and Guatemala. The distribution of endophytes depends on the host itself and the type of endophyte, and there are also differences across different organs of the host (Ottesen et al., 2013; Zheng et al., 2014; Wang et al., 2015). Gottel et al. (2011) compared the bacterial (and fungal) microbiota of mature poplar (*Populus deltoides*) trees using 16S ribosomal RNA (rRNA) gene pyrosequencing and revealed highly different endophytic bacterial communities in the root compared to the rhizosphere soil. Many endophytes can complete their own life cycle without relying on plants, and endophytes are very different across the different growth and developmental environments of plants (Kiers and Heijden, 2006), showing a large degree of environmental dependence. Hu et al. (2009) screened endogenous antagonistic bacteria against Pinellia rot. Cao et al. (2009) isolated a kind of endophytic bacterium from a Solanum plant that can be used to remove Cd^2^^+^ from the soil to avoid heavy metal pollution. Bacterial microbiota may improve nutrient bioavailability and transport from the soil, increase host tolerance to biotic stressors, promote stress resistance, and influence crop yield and quality (Beckers et al., 2017). In return, the host plant provides a habitat and a constant supply of energy and carbon sources to the microorganisms (Mendes et al., 2011; Bulgarelli et al., 2012).

Bacterial communities can live in many tissues of a plant, including the soil-root interface (rhizosphere/rhizoplane), the plant inner tissues (root, stem, and leaf endosphere), and the air-plant interface (phyllosphere environment), which provide specific biotic and abiotic conditions for the residing bacterial communities. Research on plant endophytes has become a popular research topic in the fields of botany, plant protection, pesticides, pharmacy, and Chinese medicine, and it has broad exploration and application potential in the fields of agriculture and forestry (Vandenkoornhuyse et al., 2015). Radish, a cruciferous crop, is an important traditional vegetable in China and is also widely planted around the world (Li et al., 2020). Endophytes can inhibit pathogens and improve plant resistance through competition, antibiotics and cross protection. Breeding new varieties with high yield, disease resistance, low-temperature tolerance, and suitability for cultivation in late autumn or greenhouses in winter is an important scientific research direction for endophytes in radish crops.

In this study, the V5–V6 region of the 16S rRNA gene in endophytes and rhizosphere bacteria from white radish was sequenced by Illumina-MiSeq. The bacterial endophyte communities in the rhizosphere and different radish inner compartments in different cultivars and environments were analyzed. The main influencing factors of the endophyte community in the plant were also explored to understand the resource distribution of endogenous bacteria in the different compartments and to provide a research basis for further exploration of the ecological function and interaction mechanism of microorganisms and plants.

## 2. Materials and Methods

### 2.1. Sampling Method

The study site was located at the Pingdingshan Academy of Agricultural Sciences in Chinese Henan Province (Latitude: 33°34′N; Longitude: 113°03′E). Two varieties of white radish, Pingqing No. 1 and Pingfeng No. 5, which had strong upright growth characteristics after systematic cross breeding, were screened as the research objects. They are also excellent varieties resistant to downy mildew, viral disease and black rot and can be planted in both open fields and greenhouses. Samples of the abovementioned double varieties were collected randomly from four integrated plots, including two open fields and two greenhouses, where unified agronomic management measures were adopted for fertilization and irrigation. In the initial stage, organic fertilizers were mainly used, and then nitrogen, phosphorus, and potassium compound fertilizers were supplemented. According to the soil conditions, the watering frequencies of the four plots were also similar, especially the leaf growth period and underground tuber growth period. The area of each of the abovementioned plots was over 900 m^2^, and other endangered or protected species were not identified.

On December 23, 2019, a total of 60 microbial samples that represented different cultivation conditions and radish varieties from these five different compartments (radish flesh, leaf, epidermis, root, and rhizosphere soil) were sampled from the abovementioned 4 plots before harvesting the white radish. It needs to be further clarified that the samples corresponding to each compartment of different radish varieties under the specific cultivation conditions had three replicates.

When sampling rhizosphere soil, the radish was directly pulled up to the ground using hands with sterile gloves. After shaking off the loose soil around the fibrous roots, the samples of rhizosphere soil that were thinly attached to the surface of fibrous roots were peeled off with a sterile swab and packed into sterile plastic bags to avoid contamination (Kobayashi et al., 2015). Collection of microorganisms at the phyllosphere: The leaf samples were put into a marked sterile sealed polyethylene bag and brought back to the laboratory in a portable icebox. The impurities on the surface of the vegetation were first cleaned with deionized water and then cut to 0.5 cm with aseptic scissors and placed in a 500 mL conical bottle. Then, 100 mL of PBS solution (10 mmol·L^−^^1^) at pH 7.2 and steel ball particles were added. The solution was oscillated with a constant temperature culture oscillator for 30 min and washed in an ultrasonic cleaner with 40 W power for 10 min so that the microorganisms around the leaf were separated from the plant; the solution was then oscillated for another 30 min. After shock, the bacterial liquid was centrifuged, and the sediment at the bottom of the centrifuge tube was preserved for subsequent sequencing (Zhu et al., 2018).

Undamaged samples of radish leaves, fibrous roots or abnormal fleshy roots were collected and rinsed more than three times with distilled water (Zhu et al., 2020). Plant samples, including three replicates, were collected randomly from radish leaves, fibrous roots, flesh and epidermis with a sterilized scalpel; samples were collected from different varieties and growth conditions. The mass of all samples, including the rhizosphere soil, was more than 5 g (part was used for the measurement of soil physicochemical properties), and the samples were packed into sterile plastic sampling bags and put into containers with ice at −20° immediately after on-site collection. After being sent to the lab, the collected soil samples were sieved through a 4 mm filter, and the radish tissues were ground by an MP FastPrep-24 5G homogenizer. Then, the samples were stored at −80°C before DNA extraction.

### 2.2. DNA Extraction and Amplicon Selection

Bacterial DNA was extracted from the rhizosphere soil, roots, leaves, epidermis, and radish flesh samples using the FastDNA^®^ SPIN Kit for Soil (Mpbio Bio-tek, USA) according to the manufacturer's instructions. The concentration and purity of DNA samples were determined by a NanoDrop 2000 ultraviolet-visible spectrophotometer (Thermo Scientific, USA). DNA samples were also electrophoresed on a 1% agarose gel to further assess their quality and integrity. In this experimental research, we chose the primers 799F (5′-AACMGGATTAGATACCCKG-3′) and 1193R (5′-ACGTCATCCCCACCTTCC-3′) to amplify the V5-V6 hypervariable region of the bacterial 16S rRNA encoding gene, which can effectively avoid the interference of chloroplasts in plants. The V5–V6 hypervariable regions of the bacterial 16S rRNA gene were amplified with the primers 799F and 1193R with barcodes (AGTCAC) according to a previously reported method (Bram et al., 2016; Sun et al., 2022). The PCR system included 4 μL of 5× FastPfu buffer, 2 μL of 2.5 mM dNTPs, 0.8 μL of forward primer (5 μM), 0.8 μL of reverse primer (5 μM), 0.4 μL of FastPfu DNA Polymerase, 0.2μL of BSA, 10 ng of template DNA, and ddH_2_O up to 20 μL. The PCR parameters were as follows: initial denaturation at 95°C for 3 min; 27 cycles of denaturing at 95°C for 30 s, annealing at 55°C for 30 s and extension at 72°C for 45 s; and a final extension at 72°C for 10 min. The PCR product was purified using an AxyPrep DNA Gel Extraction Kit (Axygen Biosciences, USA), and the PCR product was detected and quantified with a Quantus^*TM*^ Fluorometer. According to the sequencing requirements of each sample and the standard protocols from Majorbio Bio-Pharm Technology Co., Ltd. (Shanghai, China), purified PCR products were pooled in equimolar amounts and paired-end sequenced (2 × 300 nt) on an Illumina MiSeq platform (Illumina, San Diego, USA).

### 2.3. Sequence Quality Control and Analysis

Fastp software was employed to perform quality control on the original sequencing data (https://github.com/OpenGene/fastp), and FLASH (http://www.cbcb.umd.edu/software/flash, version 1.2.7) software (Chen et al., 2018) was used for sequence splicing. The taxonomy assignment of each sequence was obtained by aligning against the Silva 16S rRNA database (release 138) through the RDP classifier (http://rdp.cme.msu.edu/, version 2.2) with a confidence threshold of 70%.

### 2.4. Statistical Analysis

The rarefaction curve was performed at the operational taxonomic units (OTU) level with normalized OTU abundance data at 97% similarity according to the index calculated by Mothur. The difference in the alpha index value was evaluated using the Wilcoxon rank-sum test. Through ANOSIM analysis based on Bray-Curtis, the differences between groups were tested to determine whether the grouping was meaningful. Qiime calculated beta diversity distance matrix, R “vegan” software package for Non-metric multidimensional scaling analysis (NMDS) and mapping. At the same time, a Venn diagram was used to compare the shared and unique bacterial flora in different compartments at both the phylum and genus levels. The pie chart shows the distribution of bacteria in different compartments at the phylum level. The sample-species relationship diagram visualized the correspondence between samples and species, and the distribution proportion of dominant species in different groups was plotted by LefSe (LDA > 4.0). Microbial source analysis was performed by SourceTracker (Knights et al., 2011) of R software. Cooccurrence network analysis was used to show the distribution between samples and species. Network analysis was performed by Molecular Ecological Network Analysis Pipeline (MENAP) (http://ieg4.rccc.ou.edu/mena/), network diagram was drawn by Gephi.

Depending on the community abundance data in the sample, significant differences at the phylum and genus levels were evaluated by Kruskal-Wallis *H*-test and Wilcoxon rank-sum test (two groups). *P*-values were corrected using the false discovery rate (FDR) for multiple comparisons. The data for each group were calculated and analyzed based on the average of the samples within a group, and the figures were plotted by R software (Stat package of R-3.3.1 and vegan package).

## 3. Results

### 3.1. Effects of Ecological Compartment and Cultivation Environment on Microbial Diversity

A total of 3,160,716 reads were obtained from 60 white radish and soil samples after denoising. The singletons (OTUs with only one sequence) were removed from the dataset since these singletons could be due to sequencing artifacts (Beckers et al., 2017). All sequences were clustered into 2,598 OTUs with 97% similarity. These samples all almost reached the saturation phase with a Good's coverage index ≥ 98.6%, which showed that the sequencing was reliable (Supplementary Figure 1).

**Figure 1:**
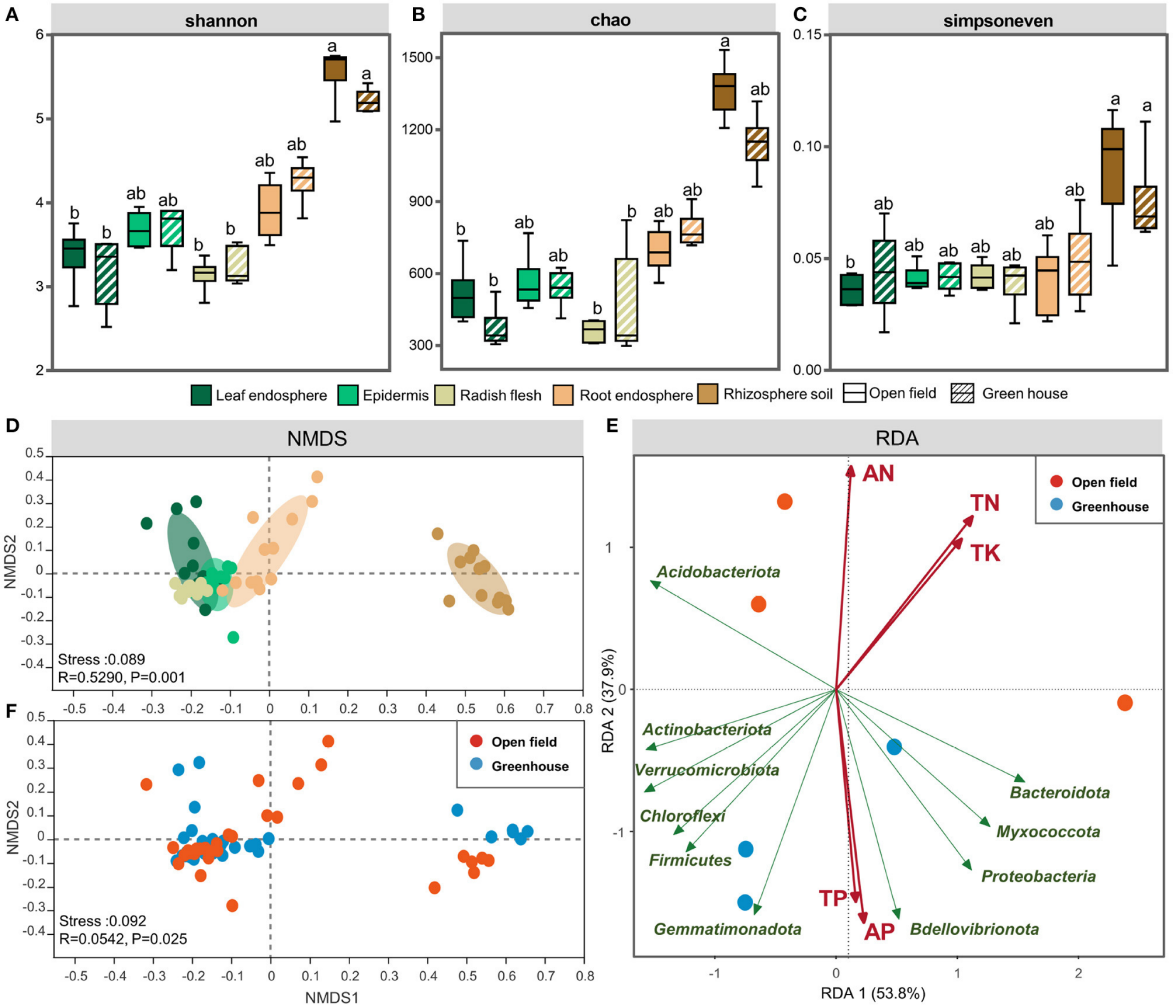
The Shannon **(A)**, Chao1 **(B)**, and Simpsoneven indices **(C)** Shannon index of the leaf endosphere, epdermis, radish flesh, root endosphere, and rhizosphere soil in open field and greenhouse. NMDS analysis of different ecological compartments **(D)** and cultivation environments **(F)**. Redundancy analysis (RDA) of the bacterial community in open field and greenhouse growth conditions **(E)**. The bar represents the median, and whiskers represent the minimum and maximum values. Data were calculated by means of a Kruskal–Wallis test. Significant differences are indicated with different lowercase letters (*P* < 0.05). The NMDS results were colored using the Bray-Curtis method. Different colors represent different plant compartments. Statistical data were analyzed by ANOSIM.

According to the sequencing data, the majority of the root endophyte samples were saturated at ~400–500 OTUs, and while saturation was reached at ~300–400 OTUs for the epidermis samples. The flesh and leaf samples closely saturated at ~100–200 OTUs. The OTUs of rhizosphere soil samples were higher than those of plant samples, reaching ~700–1,100 OTUs (Supplementary Figure 1). Statistical differences in the OTU richness, Shannon diversity index and Simpson even index were inferred from alpha diversity measures (Figure 1). These indices of rhizosphere soil samples were obviously separated from those of the radish endosphere samples (*P* < 0.01), and the rhizosphere soil samples had higher diversity and richness than the radish endosphere samples (Figures 1A,B). In terms of sample evenness, there were significant differences between rhizosphere soil samples and plant endosphere samples (including roots), and there were no significant differences among the plant tissue samples (Figure 1C).

In order to clarify the effect of grouping on endophytic and rhizosphere bacterial communities in white radish, we used PERMANOVA based on bray-curtis distance matrix to determine the main driving factors of microbial community structure. The ecological compartment was the largest explanation of bacterial distribution difference in white radish (51.8%). Among all ecological compartments of white radish, plant cultivation had the largest explanation of bacterial community differentiation (16.0–43.6%), and the underground ecological compartment had the strongest response to the environment (41.0% in root and 43.6% in rhizosphere soil, Supplementary Table 1). Compared with the higher interpretation of bacterial community distribution in the cultivation environment, the varieties did not have significant differences in the interpretation of bacterial distribution in all ecological compartments. It is worth noting that in bulbs, varieties explained the highest difference in bacterial communities (12.1%) and lowest in roots (6.5%).

At the same time, NMDS analysis also confirmed that the effect of ecological compartment on microbial clustering was greater than that of cultivation environment (Figures 1D,F). When grouping by plant compartment, at the OTU level, the results of NMDS analyses indicated that the separation between the epidermis and flesh groups was not obvious, the relative position of scattered points between the radish root and leaf groups was closer, and the rhizosphere soil group and radish root group were clearly separated from other plant endosphere groups (Figure 1D). When grouping by vertical stratification, there was a clear difference between aboveground and belowground bacterial communities. Redundancy analysis (RDA) of bacterial communities in different growth conditions showed that the samples were divided according to the environmental factors of the planting site (Figure 1E). The results of the Mantel test showed that the bacterial community structure was significantly correlated with TN (total nitrogen) and SOM (soil organic matter) (*P* < 0.05), and SOM had the highest correlation coefficient. The PERMANOVA analysis based on bray-curtis distance also proved that SOM content was the main factor affecting the underground ecological compartment. SOM had the highest explanatory power (38.4–40.3%, Supplementary Table 3) for the difference between groups of white radish cultivated in facilities and field. For the leaves and epidermis of aboveground ecological compartment, potassium had the highest explanatory power for the difference between the two compartments in different cultivation environments, which was 25.0 and 18.2%, respectively. In the stem, available potassium explained the highest amount of group (23.3%).

**Figure 2:**
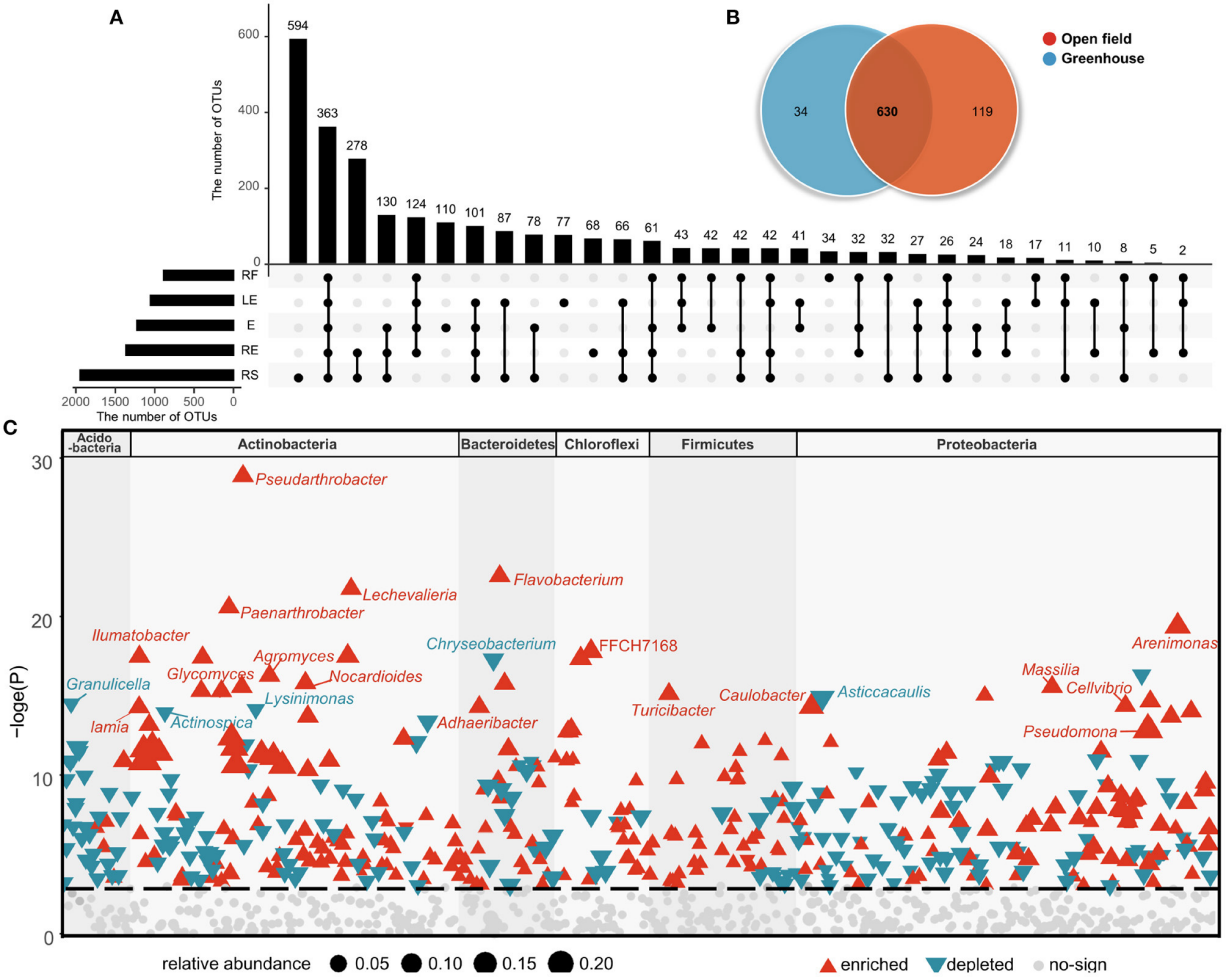
Unique and shared OTUs present in the different ecological compartments **(A)** and cultivation environments **(B)**. OTUs changes significantly in field cultivation compared to greenhouse cultivation **(C)**. Red represents OTU that is significantly enriched, blue represents OTU that is significantly depleted, and the different blocks represent phylum to which OTU belongs.

Then, we compared the community structure of the five compartments and cultivation environments at the OTU levels. At the OTU level, there were 363 OTUs shared by all groups, but different ecological compartments also featured unique bacterial flora. A total of 594 OTUs existed only in the rhizosphere soil, 110 OTUs existed only in radish epidermis, and the number of unique OTUs in the radish flesh was the lowest at 34. These data indicate that the bacterial flora were more abundant in the belowground environment than in the aboveground ecological compartments (Figure 2A). For both fields and greenhouses, the total number of OTUs is 630, while the field has 119 unique OTUs, far more than the greenhouse has 34 unique OTUs (Figure 2B).

### 3.2. Analysis of Microbial Community Composition in Different Ecological Compartments and Cultivation Environments

**Figure 3:**
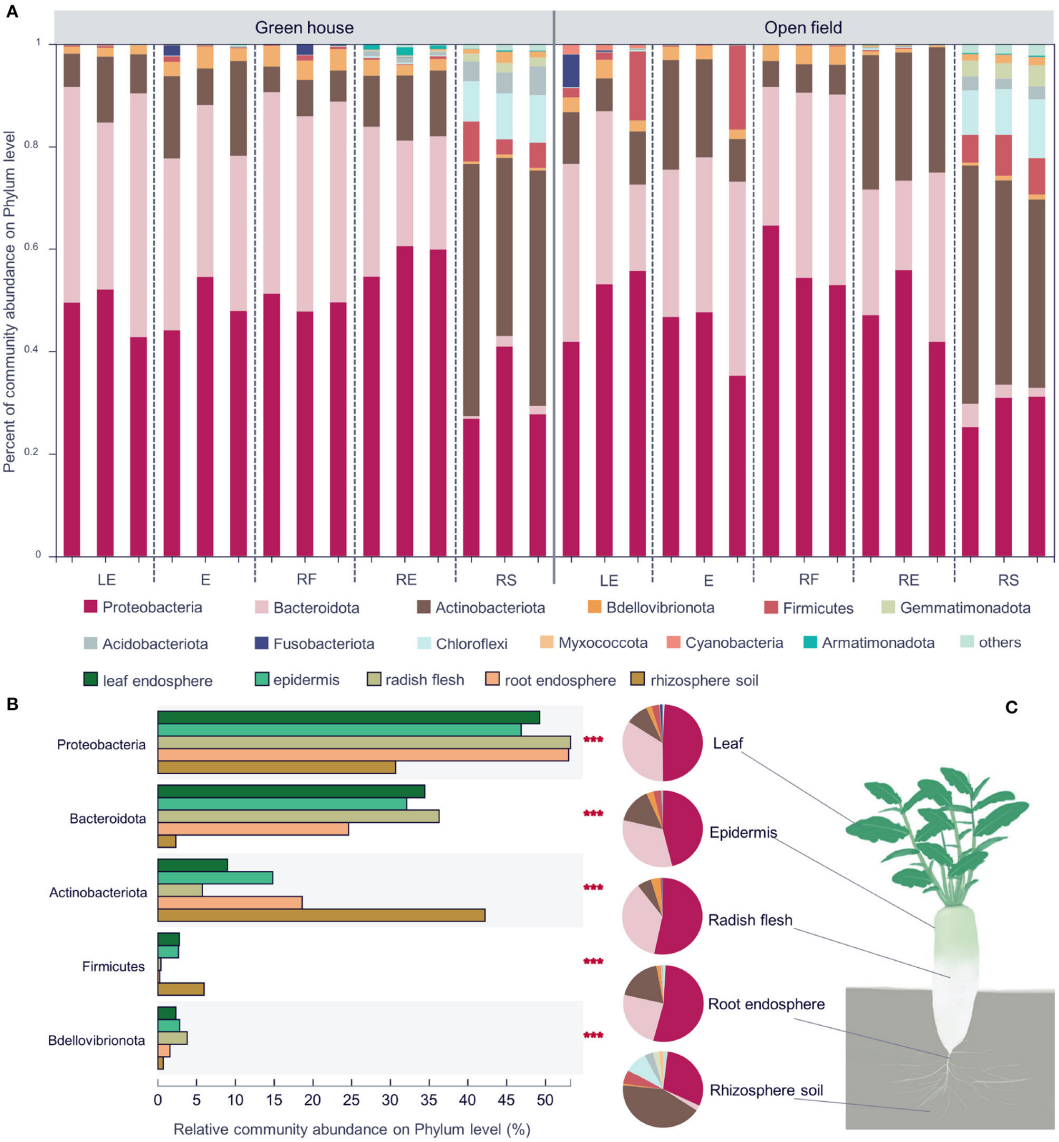
Community composition of different white radish ecological compartments at the phylum level **(A)**. Significant differences in the abundances of the top five phyla **(B)**. The major contributing phyla of rhizosphere soil, root endosphere, radish epidermis, leaf endosphere, and radish flesh are shown in different colors. Pie chart of different compartments. Different colors indicate different phyla **(C)**, and the area of the pie indicates the percentage of the taxa. Significant differences in the different groups were evaluated with the Kruskal–Wallis *H*-test, *n* = 12, in each group. LE, leaf endosphere; E, epidermis; RF, radish flesh; RE, root endosphere; RS, rhizosphere soil. ***P < 0.001.

There were 28 bacterial phyla, 73 classes, 195 orders, 361 families, 772 genera, and 1,441 species found across all samples. Among them, there were 10 phyla and 48 genera with abundances of more than 1%. The high uniformity within the group indicates (Figure 3A) that the sampling data is accurate and can effectively represent the bacterial differences between groups. *Proteobacteria* and *Bacteroidetes* were the two most abundant bacteria in the four different compartments of radish, and both *Proteobacteria* and *Actinomycetes* were the two most abundant bacteria in the rhizosphere soil group (Figures 3B,C). In all groups, the total abundance of the dominant phyla, including *Proteobacteria*, *Bacteroidetes*, and *Actinomycetes*, exceeded 90% entirely, similar to previous research (Maida et al., 2016; Sun et al., 2022). In the rhizosphere soil sample, *Bacteroidetes* showed the least abundance (2.3%), and *Actinomycetes* (42.2%) showed the highest abundance. The abundances of *Actinomycetes* in the other groups were ~5.7–18.6% (Figure 3C), and *Bdellovibrionota* had a higher abundance in the plant endosphere samples. The top five most abundant bacterial phyla all showed significant differences among the radish compartments. *Chryseobacterium*, *Flavobacterium*, *Cellvibrio*, *Brevundimonas*, and *Pseudomonas* had higher abundances in radish compartments at the genus level. *Nocardioides* had higher abundances in the rhizosphere soil groups.

In the shared OTUs, we analyzed the microorganisms that found significant changes in the two environments, and analyzed the bacterial genera that were up-regulated and down-regulated compared with those in the greenhouse (Figure 2C). In the open field, the abundance of *Pseudarthrobacter*, *Flavobacterium*, *Lechevalieria*, *Paenarthrobacter*, *Arenimonas* were higher than those in the greenhouse, and the abundance of *Chryseobacterium*, *Granulicella*, *Asticcacaulis*, *Lysinimonas*, *Actinospica* were lower (Figures 2C, 3A). In white radish leaves, the abundance of *Pseudomonas* in the open field was also significantly higher than that in the greenhouse, while the abundances of *Chryseobacterium*, *Variovorax* and *Sphingomonas* in the greenhouse were significantly higher than those in the open field. Among the endophytes of white radish roots, seven genera exhibited significant differences between the greenhouse group and open field group: *Variovorax*, *Brevundimonas*, *Dyella*, *Streptomyces*, *Cellvibrio*, *Caulobacter* and a genus of *Comamonadaceae*. In the open field group, the abundances of *Variovorax*, *Streptomyces*, and *Caulobacter* were significantly higher than those in the greenhouse group.

### 3.3. Biomarkers Analysis in Different Ecological Compartments and Cultivation Environments

**Figure 4:**
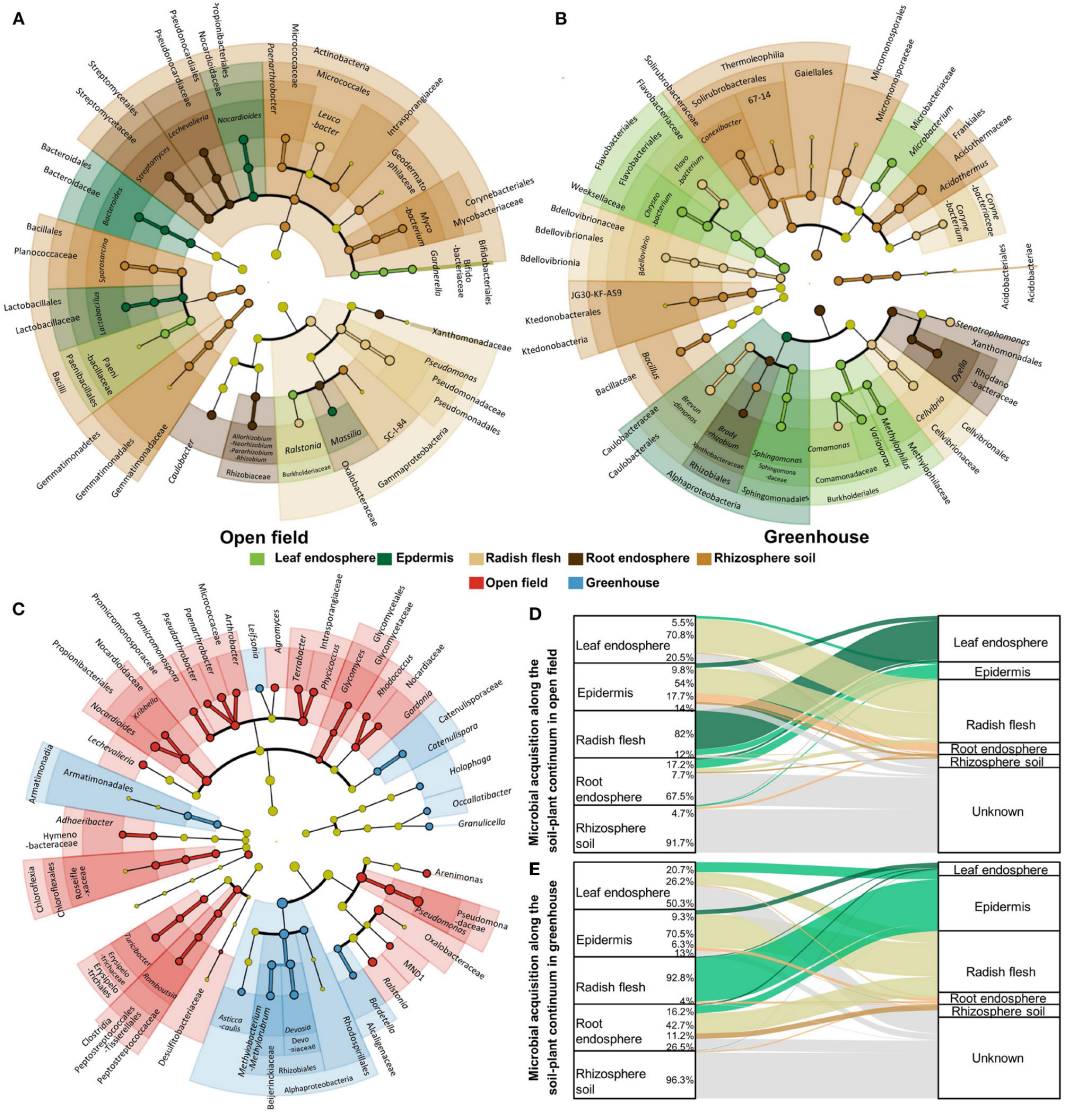
Biomarker analysis for each ecological compartment in open field **(A)** and greenhouse **(B)**, and biomarker analysis for both field and greenhouse environments **(C)**. Different colors indicate different groupings, showing biomarkers from class level to genus level from inside to outside, and light yellow indicates the absence of biomarkers in this classification. Microbial source analysis of ecological compartments in open field **(D)** and greenhouse **(E)**.

We analyzed the biomarkers in different ecological compartments in open field and greenhouse cultivation. Biomarkers were found in one ecological compartment, but relatively low in other compartments. There are more biomarkers in underground compartments, which is related to the high community richness and high species diversity in underground compartments. The number of biomarkers in the epidermal core of the aboveground septum was lower, which was more obvious in the greenhouse. In the greenhouse, only one biomarker was Alphaproteobacteria. In the field, there are more biomarkers in the epidermis, including *Nocardioides*, *Bacteroides*, and *Lactobacillus* at the genus level, and Lactobacillales, Bacteroidales, and Propionibacteriales at the Order level. In contrast, the number of biomarkers in leaves in greenhouses is much higher than in fields where only Gardnerella is at the genus level (Figures 4A,B). In addition to the biomarker analysis of ecological compartment, we also carried out biomarkers for the two cultivation environments (Figure 4C). The results showed that there were more biomarkers in the field, and there were biomarkers such as *Granulicella*, *Occallatibacte*r, *Holophaga*, *Catenulispora*, *Catenulisporaceae*, *Leifsonia*, *Asticcacaulis*, *Methylobacterium*−*Methylorubrum* in the genus level greenhouse.

We analyzed the microbial sources of ecological compartments in the fields and facilities (Figures 4D,E). There were significant indigenous differences in the assembly process of microorganisms in the non-cultivated environment, especially in the leaves and stems. 70.8% of the microorganisms in the leaves of the fields came from the stems, while only 26.2% of the microorganisms in the leaves of the greenhouse came from the stems, and most (50.3%) sources were unknown. In the stem of radish in greenhouse, 92.8% of the microorganisms were from the epidermis, while in the field, only 12% of the microorganisms were from the leaves (82%). The composition of microorganisms in roots was also different. The microorganisms in roots in greenhouses were the same source as the outer epidermis of radish, while the main source of microorganisms in roots was unknown in the field.

### 3.4. Effect of Ecological Compartment on Microbial Interaction Network

**Figure 5:**
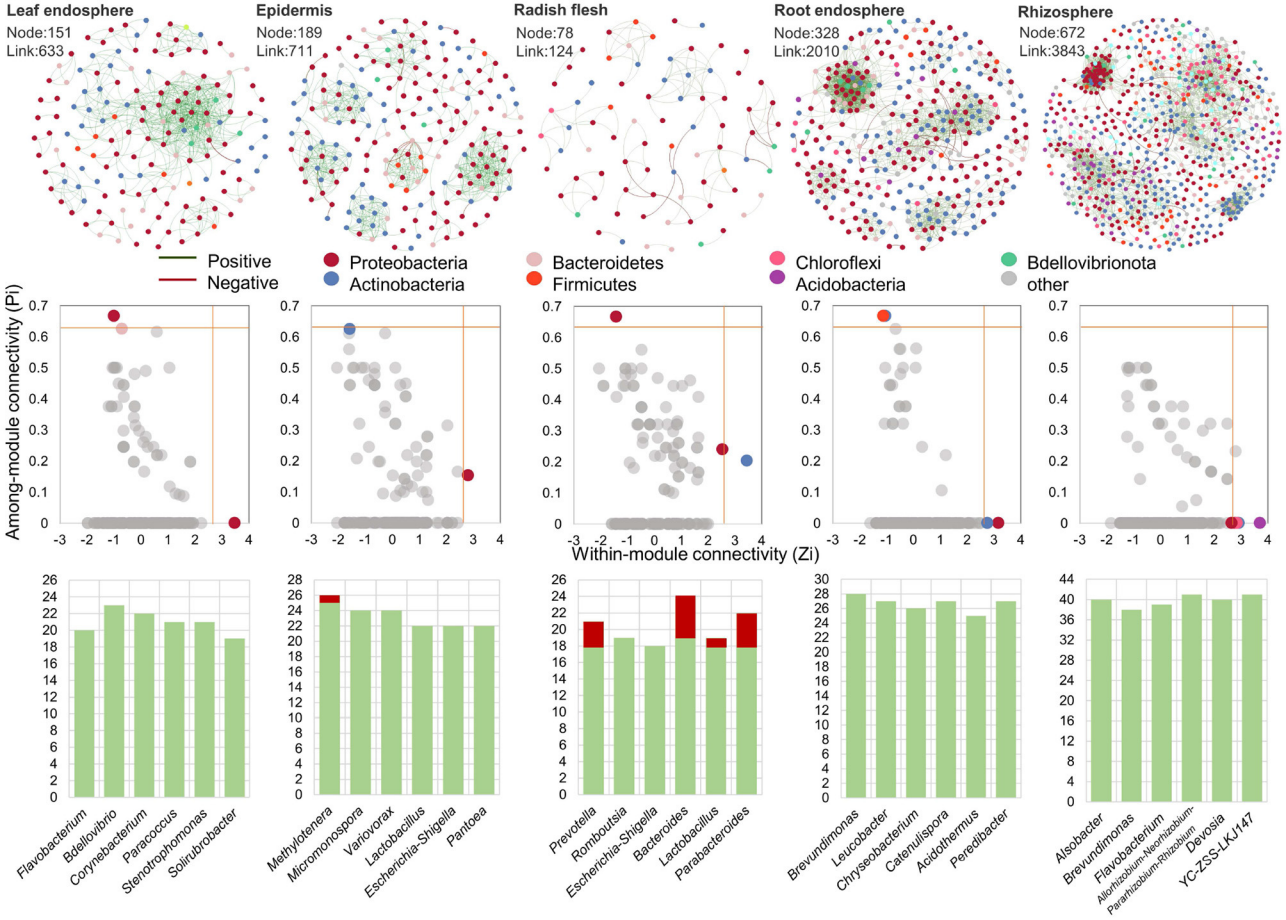
Microbial co-occurrence network diagram in leaf endosphere, radish epidermis, radish flesh, root endosphere, and rhizosphere soil. The distribution of central nodes in leaf endosphere, radish epidermis, radish flesh, root endosphere, and rhizosphere soil. The edge interaction type of genus with connectivity in the top six in different groups.

In order to further analyze the effect of ecological compartments on the relationship between leek species, OUTs with the number of more than six in each sample were selected for bacterial community co-occurrence network analysis in this study. In addition, we analyzed the interaction of microorganisms in the ecological compartments of radish and the key species that played the main connecting hub in different microbial networks. The cooccurrence network was used to display the cooccurrence relationship of species in different samples at the OTU level. The nodes in the network represent sample nodes or species nodes, and the line represents the interaction between OUTs (Figure 5). The modularity of all microbial networks was more than 90%, and the network hubs which played a key role in the whole network structure were detected in five ecological compartments. The number of module hubs in the underground ecological compartment was the largest, with a total of 12 identified, while only 3, 2, and 3 module hubs were identified in the leaves, epidermis, and stems, respectively. We further analyzed the species information of these 20 module hubs, and it was Proteobacteria that played the role of connecting center in the microbial network of the aboveground ecological compartment, followed by Bacteroidetes and Actinobacteria. In the underground ecological compartment, the main key species are Actinobacteria, followed by Bacteroidetes, Firmicutes, Armatimonadota, Proteobacteria, Acidobacteria, and Chloroflexi. We also analyzed the interaction patterns of the top six genera. The results showed that the active genera in the five ecological compartments were strongly positive interaction.

## 4. Discussion

### 4.1. Effects of Ecological Compartment and Greenhouse on the Community Structure of Endophytic Bacteria, Leaf-Surrounding Microorganisms, and Rhizosphere Microorganisms in Radish

In this study, 16srRNA was used to identify the microorganisms in each sample, and the specific changes of bacteria in different ecological compartments of radish in protected cultivation and field cultivation were analyzed. The results showed that there were significant differences in the diversity and community structure of endophytic bacteria, leaf bacteria, and rhizosphere bacteria in radish between ecological compartments and cultivated environment (Figure 1). There were also significant differences in the co-occurrence network of bacteria between ecological compartments. For the ecological compartment, the underground ecological compartment has high microbial diversity and richness, and because of the complex soil environment (Roesch et al., 2007), the richness and diversity of the rhizosphere soil sample group are the highest. The abundance and diversity of endophytic bacteria in stem ecological compartment were lower than those in other ecological compartments. The stem ecological compartment was located in the interior of radish without direct contact with the outside world, and the formation of radish tuber was later than that of root. Therefore, we believe that the bacterial community follows the gradient distribution from soil to root to stem (Shi et al., 2021), which indicates that root is also involved in the construction of the transmission channel between soil bacterial community and endophytic bacteria in other plant components. Regarding microbial uniformity, there was no significant difference among the endogenous samples corresponding to different radish compartments, but they were all significantly different from the rhizosphere soil samples (Figure 1). The rhizosphere soil-root interface acts as a selective barrier, and the endophytic competence of colonization is limited to specific bacterial species. Microorganisms colonizing plants were significantly different from those colonizing rhizosphere soil; however, these microorganisms were also related to each other because the soil around the root system is also influenced by the physiological activities of plants.

In this study, nitrogen, soil organic matter (SOM) content and potassium content significantly affected endophytic bacteria communities in leeks from different growth conditions (Figure 2B). This conclusion is consistent with previous studies (Ishida et al., 2009; Lauber et al., 2009; Shakya et al., 2013). In particular, studies in forests along a continuous transect 80 m from the coastline show that vegetation and microbial communities along the transect are strongly correlated with soil organic matter content (Merila et al., 2010). Some research has shown that the composition of plant microbes depends more on the type of soil (environment) than on the plant genotype itself (Davide et al., 2012; Mendes et al., 2013; Vandenkoornhuyse et al., 2015). Compared with field cultivation, radish soil planted in greenhouse received more organic and inorganic fertilizers, and the contents of total nitrogen and organic carbon were significantly higher than those in field soil. However, excessive fertilization and the use of pesticides may lead to the decrease of soil diversity in greenhouse. Our study also confirmed this view. Compared with field cultivation, the bacterial evenness, richness, and diversity of rhizosphere soil in greenhouse were lower. However, in the root and epidermis, the results were completely opposite. Greenhouse cultivation not only improved the bacterial alpha diversity index in the root and epidermis, but also changed the bacterial community structure in the root and epidermis, and enriched the bacteria promoting plant growth (Iliev et al., 2021) such as *Bradyrhizobium* and *Dyella* in the root.

Niche largely explains the changes in bacterial community structure (Figure 1D), highlighting the importance of niche in driving bacterial community composition and function in soil-plant continuum. It was found that the clustering of endophytic bacteria was different with compartments, and the overground and underground parts were obviously grouped into one group respectively. Plants often distribute many nutrients to the roots during their growth, and release a large number of nutrients and energy substances such as monosaccharides, polysaccharides, organic acids, phenolic compounds, amino acids, and proteins (Reinhold-Hurek et al., 2015) to the surrounding environment through their roots, which attract a large number of microorganisms. This phenomenon is more obvious when host plants are infected by pathogens. It caused changes in the internal and external environment of plants and different ecological compartments, and promoted the colonization and growth of specific endophytes. The rhizosphere microbial community is abundant in soil, and some bacteria can move actively or passively and eventually colonize plants (Hardoim et al., 2008; Stéphane et al., 2010; Sun et al., 2022).

### 4.2. Ecological Compartment and Greenhouse Affected the Enrichment of Radish Biomarkers

Generally speaking, the recruitment of microorganisms in plant roots is divided into two processes: the first step is to recruit microorganisms near the roots, and the second step is to invade the roots by specific microbial species (Hardoim et al., 2008; Stéphane et al., 2010). Our study found that the microbial groups from rhizosphere in the root endosphere of the open filed decreased, indicating that some microorganisms initially recruited to the rhizosphere were bound to the root surface and selectively filtered by the roots, which significantly affected the microbial community assembly in the roots (Figure 4). Endophytic bacteria also come from microorganisms in the air. We found that 82% of the bacteria in the stems of the field came from the leaves, while the main source of bacteria in the stems of the greenhouse was the epidermis (92.8%). 20.5–50.3% of the leaves were unknown, and we speculated that this part came from the air. There were differences in the proportion of bacteria from soil in rhizosphere between the greenhouse and the field. Compared with 96.3% of bacteria from soil in the greenhouse, 91.7% of bacteria from soil in the field. These results indicated that soil and rhizosphere communities were more sensitive to soil environmental changes, while rhizosphere communities had less response to environmental changes, which might be due to the fact that root fineness provided a more stable environment for microorganisms (Zhong et al., 2022).

There are more independent species in open field cultivation, and some genera of Actinobacteriota, Chloroflexi, and Firmicutes are significantly up-regulated in the open field (Figures 2, 3). Compared with greenhouse, the water content and soil nutrients in field cultivation are gradually reduced, indicating that open field cultivation is facing higher environmental pressure. Soil microbial community composition and function are usually related to SOC, which is mainly limited by soil carbon in field soil. In addition, temperature disturbance has always been considered as an important environmental indicator affecting microbial activity (Zander et al., 2017). The winter temperature in protected cultivation is much higher than that in field, and there is no rapid “regulation ability” in soil. There are high heterogeneity and variability in soil environmental factors, such as seasonal or periodic changes of temperature and soil moisture (López-Mondéjar et al., 2015).

The biomarkers of radish epidermis cultivated in the field were *Nocardioides* and *Bacteroides*, which were involved in organic decomposition and polysaccharide metabolism (Qiu et al., 2018; McKee et al., 2021). Biomarkers of the epidermis also include *Massilia*, which has the functions of dissolving phosphorus, degrading phenanthrene, and improving salt tolerance in crops (Krishnamoorthy et al., 2016; Lou et al., 2016). *Massilia* grew faster for nutrient-rich bacteria and responded significantly to fertilization, suggesting higher sensitivity to competition and changes in nutritional status (Suding et al., 2005; Barbosa et al., 2014). According to the strong phosphorus solubility characteristics of *Massilia* (Krishnamoorthy et al., 2016; Lou et al., 2016), and the study found that the abundance of *Massilia* was positively correlated with the activity of phosphate-solubilizing enzymes (Cardinale et al., 2019). Therefore, we believe that in field cultivation with low nitrogen content and organic matter content, *Massilia* as a biomarker in the epidermis can provide more effective phosphorus for radish, and the increase in the relative abundance of *Massilia* may also be a potential biological pathway to improve the aboveground biomass and yield of radish.

Greenhouse cultivation through reasonable management measures can reduce the impact of UV on plants, and the leaf surface is characterized by much harsher conditions, such as oligotrophy, exposure to UV radiation and desiccation (Knie et al., 2010; Atamna-Ismaeel et al., 2012; Vorholt, 2012). In our study, it was found that the number of biomarkers in the field leaves was also significantly decreased (Figures 4A,B). However, the radish leaves cultivated in greenhouse had *Microbacterium* that promoted the conversion of insoluble potassium to soluble potassium (Zhang and Kong, 2014), *Chryseobacterium* and *Sphingomonas* that promoted the decomposition of organic matter (Dahal et al., 2021), and *Variovorax* that promoted growth (Jing et al., 2015). The endophytes in the radish flesh, leaf and root compartments were significantly different between greenhouse and open field cultivation. For example, the abundances of *Pseudomonas* and *Streptomyces* in the open field group were higher than those in the greenhouse group. Plant microbiota can be seen as a component of plant defence (Tan et al., 2017). The difference may be part of their survival mechanism for adapting to different humidities and temperatures.

### 4.3. *Raphanus sativus*—Specific Microbial Community Improves Stability of Underground Compartment

The microbial co-occurrence network in the underground compartment has much higher network diameter, node number and connection number than that in the aboveground compartment (Figure 5), and has a larger scale of co-occurrence network and more complex and stable network structure. With the upward movement of the ecological compartment, the node hub role of Actinomycetes in the microbial co-occurrence network decreases, and the hub role of Proteobacteria increases. In addition, in a specific network, higher modularity represents a relatively stable community. In our study, the modularity level of epidermis is the highest (80.7%), while that of leaf is the lowest (50.8%). This trend is proportional to the proportion of negative interactions between ecological compartments. Therefore, we believe that the modularity level is related to the interaction between microbial communities (Herren and McMahon, 2017). Modularization can reduce the impact on its own modules by limiting biological groups, and prevent the disappearance of biological groups from affecting other parts of the network.

The positive and negative correlation between microorganisms is also an important indicator reflecting the microbial network structure. The results showed that most of the interactions among microorganisms in ecological compartments were positively correlated (90.2–99.5%), indicating that most microorganisms were inclined to co-occurrence rather than co-exclusion. The study of microbial interaction network shows that when there is a high proportion of negative and positive correlation between microbial groups, the ecological network is more stable and the ability to cope with environmental changes is stronger, which is related to the negative interaction that reduces the co-oscillation of disturbed communities (Coyte et al., 2015). In this study, it is found that there will be a high proportion of negative interactions between underground ecological compartments, which may be related to the more complex environment in which underground ecological compartments need to respond to environmental changes. Studies have also shown that environmental stress undermines the stability of microbial community networks, as positive correlation communities occur more frequently under higher environmental stress (Stouffer and Bascompte, 2011). It is worth noting that in stems, the top six genera with connectivity have higher negative interactions, which may be related to the competition for niche resources within plants. This competition is generally divided into two types: one is that microorganisms compete with each other due to the lack of sufficient food, resulting in a high proportion of negative correlation (Zhong et al., 2022); second, niche overlap occurs when multiple microorganisms utilize the same resource in the stem of radish. Therefore, microorganisms with similar ecological characteristics or similar survival requirements are more prone to niche overlap in the community. The value of niche overlap is proportional to the strength of competition for resources. If this overlap exists in the case of full saturation of environmental capacity, it will lead to competitive exclusion (Clavel et al., 2011; Duan et al., 2017).

Since they are closely related to other microorganisms, key species in the community may play a unique and critical role in maintaining community structure and stability, which can drive the formation of microbial communities (Banerjee et al., 2016). Therefore, by analyzing the intra-module connectivity and inter-module connectivity of nodes (OTUs) in different ecological compartment ecological networks, the key species in different ecological compartment ecological networks are identified. In the networks with different ecological compartments, the connectivity between modules of most OTUs is <0.62, and the connectivity within modules is <2.5. In other words, the connectivity between modules and within modules of most OTUs is not high, belonging to peripheral nodes, which is similar to the distribution of different species among ecological compartments. The main connection hub between aboveground compartments is Proteobacteria, and the main connection hub between underground compartments is Actinobacteria (Figures 4, 5). It has been pointed out that the microbial network structure needs to rely on some very active OTUs for information exchange or intermediate metabolites to maintain a large and complex modular structure. More modular hubs can improve the exchange efficiency of microbial communities. When encountering environmental disturbance, the lack of key species may damage the stability of the community, and cause great changes in the composition and function of the community (Banerjee et al., 2019). Therefore, microorganisms in underground ecological compartments with more module hubs and connections may be more efficient for material transmission and utilization, and at the same time, they have stronger ability to resist environmental disturbances.

In summary, we analyzed the effects of different ecological compartments and two cultivation environments on microorganisms in radish, and found that ecological compartments played an important role, which was related to the assembly mechanism of microorganisms in plants. The same facility cultivation could also improve the diversity of microorganisms in different ecological compartments of radish, and change the biomarkers that played a major role in rhizosphere microorganisms and endophytic microorganisms of radish. The sources of microorganisms in different ecological compartments of radish indicated that greenhouse changed the assembly mechanism in plants. We also analyzed the interaction network of radish microorganisms in different ecological compartments, and found that different key microorganisms play a connecting hub role in different ecological compartments. These results showed that different ecological compartments and cultivation environments had significant indigenous effects on plants, which provided a theoretical basis for the subsequent isolation and screening of endophytic bacteria.

## Data Availability Statement

The datasets presented in this study can be found in online repositories. The names of the repository/repositories and accession number(s) can be found in the article/Supplementary Material.

## Author Contributions

NS: data processing, picture drawing, paper writing, and the paper revising. YG: draft writing and sample collection. GJ: sample collection and theoretical guidance. YW: sample planting and theoretical guidance. PW: sample collection. WS, PM, YD, and ZJ: sample planting and related experimental materials maintenance. All authors listed have made a substantial, direct, and intellectual contribution to the work and approved it for publication.

## Funding

This research was financially supported by the China Agricultural University University-Industry Cooperation Science and Technology Project (GZXCZH201811002), the Beijing Natural Science Fund-Haidian Original Innovation Joint Fund (L212068), and Science and Technology Cooperation Project of China Agricultural University (PFZN202106).

## Conflict of Interest

The authors declare that the research was conducted in the absence of any commercial or financial relationships that could be construed as a potential conflict of interest.

## Publisher's Note

All claims expressed in this article are solely those of the authors and do not necessarily represent those of their affiliated organizations, or those of the publisher, the editors and the reviewers. Any product that may be evaluated in this article, or claim that may be made by its manufacturer, is not guaranteed or endorsed by the publisher.

## References

[B1] Atamna-Ismaeel N. Finkel O. M. Glaser F. Sharon I. Schneider R. Post A. F.. (2012). Microbial rhodopsins on leaf surfaces of terrestrial plants. Environ. Microbiol. 14, 140–146. 10.1111/j.1462-2920.2011.02554.x 21883799 PMC3608849

[B2] Banerjee S. Kirkby C. A. Schmutter D. Bissett A. Kirkegaard J. A. Richardson A. E. (2016). Network analysis reveals functional redundancy and keystone taxa amongst bacterial and fungal communities during organic matter decomposition in an arable soil. Soil Biol. Biochem. 97, 188–198. 10.1016/j.soilbio.2016.03.017

[B3] Banerjee S. Walder F. Büchi L. Meyer M. Held A. Y. Gattinger A.. (2019). Agricultural intensification reduces microbial network complexity and the abundance of keystone taxa in roots. ISME J. 13, 1722–1736. 10.1038/s41396-019-0383-2 30850707 PMC6591126

[B4] Barbosa E. R. Tomlinson K. W. Carvalheiro L. G. Kirkman K. de Bie S. Prins H. H.. (2014). Short-term effect of nutrient availability and rainfall distribution on biomass production and leaf nutrient content of savanna tree species. PLoS ONE 9:e92619. 10.1371/journal.pone.0092619 24667837 PMC3965441

[B5] Beckers B. Beeck M. Weyens N. Boerjan W. Vangronsveld J. (2017). Structural variability and niche differentiation in the rhizosphere and endosphere bacterial microbiome of field-grown poplar trees. Microbiome 5:25. 10.1186/s40168-017-0241-2 28231859 PMC5324219

[B6] Bram B. Michiel O. Sofie T. Sascha T. Nele W. Wout B.. (2016). Performance of 16s rdna primer pairs in the study of rhizosphere and endosphere bacterial microbiomes in metabarcoding studies. Front. Microbiol. 7:650. 10.3389/fmicb.2016.00650 27242686 PMC4865482

[B7] Bulgarelli D. Schlaeppi K. Spaepen S. Themaat E. V. Schulze-Lefert P. (2012). Structure and functions of the bacterial microbiota of plants. Annu. Rev. Plant Biol. 64, 807–838. 10.1146/annurev-arplant-050312-120106 23373698

[B8] Cao Z. Luo S. L. Zeng G. M. Xiao X. Wan Y. Feng S. (2009). Removal of CD (2+) by an endophytic bacteria sde06 obtained from *Solanum nigrum* l. Microbiology 36, 328–333. 10.13344/j.microbiol.china.2009.03.018

[B9] Cardinale M. Suarez C. Steffens D. Ratering S. Schnell S. (2019). Effect of different soil phosphate sources on the active bacterial microbiota is greater in the rhizosphere than in the endorhiza of barley (*Hordeum vulgare* L.). Microb. Ecol. 77, 689–700. 10.1007/s00248-018-1264-3 30259168

[B10] Chen S. F. Zhou Y. Q. Chen Y. R. Gu J. (2018). FASTP : an ultra-fast all-in-one FASTQ preprocessor. Front. Microbiol. 34, i884–i890. 10.1093/bioinformatics/bty560 30423086 PMC6129281

[B11] Clavel J. Julliard R. Devictor V. (2011). Worldwide decline of specialist species: toward a global functional homogenization? Front. Ecol. Environ. 9, 222–228. 10.1890/080216

[B12] Coyte K. Z. Schluter J. Foster K. R. (2015). The ecology of the microbiome: networks, competition, and stability. Science 350, 663–666. 10.1126/science.aad2602 26542567

[B13] Dahal R. H. Chaudhary D. K. Kim D.-U. Pandey R. P. Kim J. (2021). Chryseobacterium *Antibioticum* sp. nov. with antimicrobial activity against gram-negative bacteria, isolated from arctic soil. J. Antibiot. 74, 115–123. 10.1038/s41429-020-00367-1 32895493

[B14] Davide B. Matthias R. Klaus S. Emiel V. L. V. V. T. Nahal A. Federica A.. (2012). Revealing structure and assembly cues for arabidopsis root-inhabiting bacterial microbiota. Nature 488, 91–95. 10.1038/nature11336 22859207

[B15] Duan H. Zhao A. Yao Z. (2017). Analysis of wetland plant-soil relationships and population niches in Chayegang Marshland near Henghu farm in the Poyang lake region during the dry season. Acta Ecol. Sin. 37, 3744–3754. 10.5846/stxb201604050614

[B16] Gottel N. R. Castro H. F. Kerley M. Yang Z. Pelletier D. A. Podar M.. (2011). Distinct microbial communities within the endosphere and rhizosphere of populus deltoides roots across contrasting soil types. Appl. Environ. Microbiol. 77, 5934–5944. 10.1128/AEM.05255-11 21764952 PMC3165402

[B17] Hardoim P. R. Overbeek L. S. V. Elsas J. D. V. (2008). Properties of bacterial endophytes and their proposed role in plant growth. Trends Microbiol. 16, 463–471. 10.1016/j.tim.2008.07.008 18789693

[B18] Herren C. M. McMahon K. D. (2017). Cohesion: a method for quantifying the connectivity of microbial communities. ISME J. 11, 2426–2438. 10.1038/ismej.2017.91 28731477 PMC5649174

[B19] Hu X. F. Fang Q. L. Li S. X. Wu J. G. Chen J. S. (2009). Isolation and characterization of endophytic and rhizosphere bacterial antagonists of soft rot pathogen from *Pinellia ternata*. FEMS Microbiol. Lett. 295, 10–16. 10.1111/j.1574-6968.2009.01558.x 19473246

[B20] Iliev I. Apostolova E. Hadjieva N. Kostadinov K. Filipov S. Kostadinova S.. (2021). Bacterial diversity and physiological activity of lettuce (*Lactuca sativa*) rhizosphere under bio-organic greenhouse management strategies. Int. J. Environ. Sci. Technol. 10.1007/s13762-021-03831-z. [Epub ahead of print].

[B21] Ishida T. A. Nara K. Ma S. Takano T. Liu S. (2009). Ectomycorrhizal fungal community in alkaline-saline soil in northeastern china. Mycorrhiza 19, 329–335. 10.1007/s00572-008-0219-9 19104846

[B22] Jing Y. Wu Y. Wang G. Xu W. Zhang Z. Xu L.. (2015). Plant growth-promoting bacterium *Variovorax* sp. jx14 from calcareous alluvial soil: characterization and growth promotion on peanuts. Soils 47, 698–703. 10.13758/j.cnki.tr.2015.04.012

[B23] Kiers E. T. Heijden M. G. A. V. D. (2006). Mutualistic stability in the arbuscular mycorrhizal symbiosis: exploring hypotheses of evolutionary cooperation. Ecology 87, 1627–1636. 10.1890/0012-9658(2006)87[1627:MSITAM]2.0.CO;2 16922314

[B24] Knie C. Ramette A. Frances L. Carlos A. B. Vorholt J. A. (2010). Site and plant species are important determinants of the methylobacterium community composition in the plant phyllosphere. ISME J. 4, 719–728. 10.1038/ismej.2010.9 20164863

[B25] Knights D. Kuczynski J. Charlson E. S. Zaneveld J. Mozer M. C. Collman R. G.. (2011). Bayesian community-wide culture-independent microbial source tracking. Nat. Methods 8, 761–763. 10.1038/nmeth.1650 21765408 PMC3791591

[B26] Kobayashi A. Kobayashi Y. O. Someya N. Ikeda S. (2015). Community analysis of root- and tuber-associated bacteria in field-grown potato plants harboring different resistance levels against common scab. Microbes Environ. 30, 301–309. 10.1264/jsme2.ME15109 26657303 PMC4676553

[B27] Krishnamoorthy R. Kim K. Subramanian P. Senthilkumar M. Anandham R. Sa T. (2016). Arbuscular mycorrhizal fungi and associated bacteria isolated from salt-affected soil enhances the tolerance of maize to salinity in coastal reclamation soil. Agric. Ecosyst. Environ. 231, 233–239. 10.1016/j.agee.2016.05.037

[B28] Lauber C. L. Hamady M. Knight R. Fierer N. (2009). Pyrosequencing-based assessment of soil ph as a predictor of soil bacterial community structure at the continental scale. Appl. Environ. Microbiol. 75, 5111–5120. 10.1128/AEM.00335-09 19502440 PMC2725504

[B29] Li L. Zhang X. K. Zhou Z. Z. Lu C. Pan X. Deng X. Y. (2020). Comparison of extraction methods for insoluble dietary fiber of white radish and the effect of IDF on biscuits digestion. Farm Prod. Process. 38-41. 10.16693/j.cnki.1671-9646(X).2020.07.044

[B30] Long C. Liang Z. Hua Z. (2015). Research advances in the studies of plant entophytic. Biotechnol. Bull. 31, 30–34. 10.13560/j.cnki.biotech.bull.1985.2015.08.005

[B31] López-Mondéjar R. Voříšková J. Větrovský T. Baldrian P. (2015). The bacterial community inhabiting temperate deciduous forests is vertically stratified and undergoes seasonal dynamics. Soil Biol. Biochem. 87, 43–50. 10.1016/j.soilbio.2015.04.008

[B32] Lou J. Gu H. Wang H. An Q. Xu J. (2016). Complete genome sequence of *Massilia* sp. wg5, an efficient phenanthrene-degrading bacterium from soil. J. Biotechnol. 218, 49–50. 10.1016/j.jbiotec.2015.11.026 26656222

[B33] Maida I. Chiellini C. Mengoni A. Bosi E. Fani R. (2016). Antagonistic interactions between endophytic cultivable bacterial communities isolated from the medicinal plant e *Chinacea purpurea*. Environ. Microbiol. 18, 2357–2365. 10.1111/1462-2920.12911 26013664

[B34] McKee L. S. La Rosa S. L. Westereng B. Eijsink V. G. Pope P. B. Larsbrink J. (2021). Polysaccharide degradation by the bacteroidetes: mechanisms and nomenclature. Environ. Microbiol. Rep. 13, 559–581. 10.1111/1758-2229.12980 34036727

[B35] Mendes R. Garbeva P. Raaijmakers J. M. (2013). The rhizosphere microbiome: significance of plant beneficial, plant pathogenic, and human pathogenic microorganisms. FEMS Microbiol. Rev. 37, 634–663. 10.1111/1574-6976.12028 23790204

[B36] Mendes R. Kruijt M. Bruijn I. D. Dekkers E. Voort M. Schneider J.. (2011). Deciphering the rhizosphere microbiome for disease-suppressive bacteria. Science 332, 1097–1100. 10.1126/science.1203980 21551032

[B37] Merila P. Malmivaara-Lamsa M. Spetz P. Stark S. Vierikko K. Derome J.. (2010). Soil organic matter quality as a link between microbial community structure and vegetation composition along a successional gradient in a boreal forest. Appl. Soil Ecol. 46, 259–267. 10.1016/j.apsoil.2010.08.003

[B38] Ottesen A. R. Pena A. G. White J. R. Pettengill J. B. Brown E. (2013). Baseline survey of the anatomical microbial ecology of an important food plant: *Solanum lycopersicum* (tomato). BMC Microbiol. 13, 114–114. 10.1186/1471-2180-13-114 23705801 PMC3680157

[B39] Qiu J. Zhang Y. Zhao L. He Q. Jiang J. Hong Q. He J. (2018). Isolation and characterization of the cotinine-degrading bacterium *Nocardioides* sp. strain JQ2195. J. Hazard. Mater. 353, 158–165. 10.1016/j.jhazmat.2018.04.003 29665494

[B40] Reinhold-Hurek B. Bünger W. Burbano C. S. Sabale M. Hurek T. (2015). Roots shaping their microbiome: global hotspots for microbial activity. Annu. Rev. Phytopathol. 53, 403–424. 10.1146/annurev-phyto-082712-102342 26243728

[B41] Roesch L. F. Fulthorpe R. R. Riva A. Casella G. Hadwin A. Kent A. D.. (2007). Pyrosequencing enumerates and contrasts soil microbial diversity. ISME J. 13, 283–290. 10.1038/ismej.2007.53 18043639 PMC2970868

[B42] Shakya M. Gottel N. Castro H. Yang Z. K. Gunter L. Labbé J.. (2013). A multifactor analysis of fungal and bacterial community structure in the root microbiome of mature populus deltoides trees. PLoS ONE 8:e76382. 10.1371/journal.pone.0076382 24146861 PMC3797799

[B43] Shi W. Su G. Li M. Wang B. Lin R. Yang Y. Wei T. Zhou B. Gao Z. (2021). Distribution of bacterial endophytes in the non-lesion tissues of potato and their response to potato common scab. Front. Microbiol. 12:114. 10.3389/fmicb.2021.616013 33633704 PMC7900429

[B44] Stéphane C. Christophe C. Angela S. (2010). Plant growth-promoting bacteria in the rhizo- and endosphere of plants: their role, colonization, mechanisms involved and prospects for utilization. Soil Biol. Biochem. 42, 669–678. 10.1016/j.soilbio.2009.11.024

[B45] Stouffer D. B. Bascompte J. (2011). Compartmentalization increases food-web persistence. Proc. Natl. Acad. Sci. U.S.A. 108, 3648–3652. 10.1073/pnas.1014353108 21307311 PMC3048152

[B46] Suding K. N. Collins S. L. Gough L. Clark C. Cleland E. E. Gross K. L.. (2005). Functional-and abundance-based mechanisms explain diversity loss due to n fertilization. Proc. Natl. Acad. Sci. U.S.A. 102, 4387–4392. 10.1073/pnas.0408648102 15755810 PMC555488

[B47] Sun N. Wang Y. X. Chen J. H. Wang P. Z. Song W. T. Ma P. F.. (2022). Colonization and interaction of bacteria associated with Chinese chives affected by ecological compartments and growth conditions. Front. Microbiol. 13:775002. 10.3389/fmicb.2022.775002 35237245 PMC8883035

[B48] Tan Y. Cui Y. Li H. Kuang A. Li X. Wei Y.. (2017). Diversity and composition of rhizospheric soil and root endogenous bacteria in *Panax notoginseng* during continuous cropping practices. J. Basic Microbiol. 57:337. 10.1002/jobm.201600464 28060404

[B49] Vandenkoornhuyse P. Quaiser A. Duhamel M. Van A. L. Dufresne A. (2015). The importance of the microbiome of the plant holobiont. New Phytol. 206, 1196–1206. 10.1111/nph.13312 25655016

[B50] Vorholt J. A. (2012). Microbial life in the phyllosphere. Nat. Rev. Microbiol. 10, 828–840. 10.1038/nrmicro2910 23154261

[B51] Wang E. T. Tan Z. Y. Guo X. W. Rodríguezduran R. Boll G. Martínezromero E. (2006). Diverse endophytic bacteria isolated from a leguminous tree conzattia multiflora grown in Mexico. Arch. Microbiol. 186, 251–259. 10.1007/s00203-006-0141-5 16862424

[B52] Wang Z. W. Ling J. Y. Chen Y. G. (2015). Studies and biological significances of plant endophytes. Microbiol. China 42, 349–363. 10.13344/j.microbiol.china.130815

[B53] Zander A. Bersier L.-F. Gray S. M. (2017). Effects of temperature variability on community structure in a natural microbial food web. Glob. Change Biol. 23, 56–67. 10.1111/gcb.13374 27234703

[B54] Zhang C. Kong F. (2014). Isolation and identification of potassium-solubilizing bacteria from tobacco rhizospheric soil and their effect on tobacco plants. Appl. Soil Ecol. 82, 18–25. 10.1016/j.apsoil.2014.05.002

[B55] Zheng Y. K. Liu K. Xiong Z. J. Li W. J. Zhao L. X. (2014). Research progress on biodiversity of endophytic actinobacteria in medicinal plants and their bioactive substances. Chinese Tradit. Herb. Drugs 45, 2089–2099. 10.7501/j.issn.0253-2670.2014.14.025

[B56] Zhong Y. Sorensen P. O. Zhu G. Jia X. Liu J. Shangguan Z.. (2022). Differential microbial assembly processes and co-occurrence networks in the soil-root continuum along an environmental gradient. iMeta 1:e18. 10.1002/imt2.18 PMC10989781 38868564

[B57] Zhu B. Wu J. Ji Q. Wu W. Dong S. Yu J.. (2020). Diversity of rhizosphere and endophytic fungi in atractylodes macrocephala during continuous cropping. PeerJ 8:e8905. 10.7717/peerj.8905 32292655 PMC7144587

[B58] Zhu H. Li S. Y. Hu Z. Y. Liu G. X. (2018). Molecular characterization of eukaryotic algal communities in the tropical phyllosphere based on real-time sequencing of the 18s rDNA gene. BMC Plant Biol. 18:365. 10.1186/s12870-018-1588-7 30563464 PMC6299628

