# Supplementary material for: Bacterial Communities in the Endophyte and Rhizosphere of White Radish (Raphanus sativus) in Different Compartments and Growth Conditions
Source: Front Microbiol. 2022 Jun 29;13:900779. doi: 10.3389/fmicb.2022.900779 (PMC9277120; doi:10.3389/fmicb.2022.900779)
Supplement: Supplementary file 1 [file Data_Sheet_1.PDF]

Supplementary Material

0.1 Figures

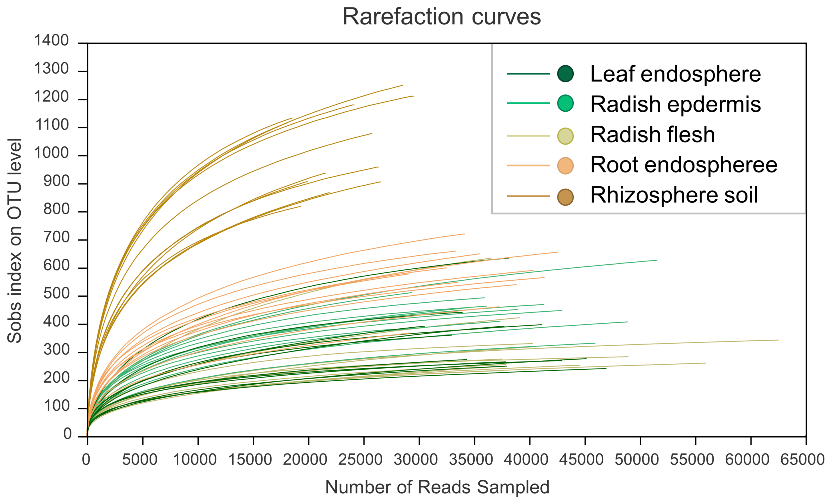

Figure S1: Rarefaction curves of radish different compartments.

Table S1. Effects of multiple biotic and abiotic factors on the microbiome assembly

Samples	Variables	White radish	
		R^2	P
All samples (n = 60)	Compartment niche	0.518	0.003
	Planting environment	0.045	0.048
	Gene type	0.008	0.873
Leaf endosphere (n = 12)	Planting environment	0.259	0.038
	Gene type	0.067	0.589
Epidermis (n = 12)	Planting environment	0.160	0.070
	Gene type	0.073	0.686
Stem endosphere (n = 12)	Planting environment	0.229	0.032
	Gene type	0.121	0.250
Root endosphere (n = 12)	Planting environment	0.410	0.008
	Gene type	0.065	0.716
Rhizosphere soil (n = 12)	Planting environment	0.436	0.004
	Gene type	0.094	0.305

The significance of different factors on microbial community dissimilarity was tested with PERMANOVA (based on bray-curtis distances).

Table S2. Physical and chemical properties of soil in open field and greenhouse

Growth condition	SOM (g/kg)	TN g/kg	TP (g/kg)	TK (g/kg)	AN g/kg	AP (mg/kg)	AK g/kg
Open field	9.731±1.130	1.088±0.089	12.715±1.677	20.200±2.825	0.272±0.086	23.034±1.797	0.295±0.058
Greenhouse	28.099±3.583	1.562±0.178	8.084±1.189	20.779±1.820	0.556±0.100	14.985±4.447	0.291±0.010

Table S3. Effects of soil physicochemical properties on microbial assembly

Enviromental parameter	All samples		Leaf endosphere		Epidermis		Stem endosphere		Root endosphere		Rhizosphere soil	
	R^2	P	R^2	P	R^2	P	R^2	P	R^2	P	R^2	P
SOM	0.043	0.033	0.232	0.031	0.156	0.052	0.226	0.013	0.384	0.002	0.403	0.002
TN	0.039	0.051	0.200	0.051	0.176	0.021	0.179	0.084	0.354	0.001	0.325	0.008
AN	0.036	0.056	0.215	0.020	0.127	0.172	0.297	0.002	0.325	0.003	0.370	0.001
AP	0.024	0.161	0.150	0.117	0.102	0.296	0.085	0.394	0.218	0.011	0.277	0.005
TP	0.023	0.198	0.162	0.089	0.118	0.230	0.137	0.198	0.211	0.024	0.244	0.028
TK	0.019	0.281	0.250	0.286	0.182	0.095	0.031	0.834	0.237	0.010	0.091	0.467
AK	0.009	0.820	0.046	0.867	0.057	0.751	0.233	0.057	0.107	0.271	0.056	0.687

The significance of different factors on microbial community dissimilarity was tested with PERMANOVA (based on bray-curtis distances).
